# No apparent increase in cases of severe acute hepatitis of unknown etiology with fulminant liver failure in children in Germany, 2022

**DOI:** 10.1002/jpr3.70083

**Published:** 2025-09-12

**Authors:** Anna‐Lisa Behnke, Achim Dörre, Julia Enkelmann, Birgit Knoppke, Working Group for Pediatric Liver Transplantation of the German Society for Pediatric Gastroenterology and Nutrition (GPGE), Ruth Zimmermann, Mirko Faber

**Affiliations:** ^1^ Department of Infectious Disease Epidemiology Robert Koch Institute Berlin Germany; ^2^ Postgraduate Training for Applied Epidemiology (PAE), Robert Koch Institute Berlin Germany; ^3^ ECDC Fellowship Programme, Field Epidemiology path (EPIET), European Centre for Disease Prevention and Control (ECDC) Stockholm Sweden; ^4^ University Children's Hospital Regensburg Regensburg Germany; ^5^ Spokesperson of the Working Group for Pediatric Liver Transplantation, German Society for Pediatric Gastroenterology and Nutrition (GPGE) Berlin Germany

**Keywords:** disease outbreaks, hepatic failure, liver transplantation, pediatric, viral hepatitis

## Abstract

**Objectives:**

In April 2022, the United Kingdom and the United States reported alarming increases in cases of severe acute hepatitis of unknown etiology in children, indicating a multicountry outbreak. We aimed to determine if Germany was affected by the outbreak.

**Methods:**

Cases were defined as patients 0–16 years with severe acute hepatitis (aspartate transaminase and/or alanine transaminase > 500 IU/l), with adenovirus detection or unknown etiology. Severely impaired liver function resulting in listing for liver transplant, undergoing liver transplant or death was defined as “fulminant” pediatric acute liver failure (pALF). We compared the 2017–2021 case numbers reported by German pediatric liver transplant centers (pLTxCs) with 05/2022–05/2023. Numbers of pediatric inpatients diagnosed with any of 12 selected hepatological ICD‐10 codes and number of liver transplants from national hospital discharge data in 2022 were compared with 2015–2021, using a two‐sample Poisson test.

**Results:**

From 5/2022–5/2023, eight pLTxCs reported nine hepatitis cases with fulminant pALF, compared to 5–14 cases annually from 2017 to 2021, and a total of 26 hepatitis cases without pALF (no baseline data available). The number of pediatric inpatients diagnosed with any of 12 selected hepatological ICD‐10 codes was 373 in 2022, compared to 333–422 annually in 2015–2021. There were 85 liver transplants in 2022, compared to 91–114 annually in 2015–2021.

**Conclusions:**

According to available data, there was no apparent increase in severe acute hepatitis cases of unknown etiology with fulminant pALF in children in Germany, 2022. We recommend implementing syndromic surveillance at pediatric emergency units and timely access to hospital discharge data.

## Abstract (highlights)

**What is Known:**

- In April 2022, public health authorities in the United Kingdom reported a steep increase in severe acute hepatitis cases in previously healthy children of whom several required liver transplants.
- There was concern that similar outbreaks could exist undetected in other countries.

**What is New:**

- Our findings indicate that, based on the available data, there was no apparent increase in cases of severe acute hepatitis of unknown etiology with fulminant liver failure in children in Germany during 2022.
- Close collaboration between public health authorities and clinicians is crucial for effective surveillance, especially when investigating conditions not previously subject to routine monitoring.

## 1 INTRODUCTION

In April 2022, the United Kingdom notified the World Health Organization (WHO) of an unexpected increase of severe acute hepatitis cases of unknown etiology in Scotland in previously healthy children younger than 10 years.^1^ As of June 2022, there were 260 cases in the United Kingdom, of which 12 cases required a liver transplant.^2^

Following the alert, the United States, several European Union, European Economic Area (EU/EEA), and other countries reported suspected cases, indicating a potential multicountry hepatitis outbreak of unknown etiology. Generally, severe acute hepatitis of unknown etiology is a rare pediatric condition and data on epidemiology and underlying causes are scarce^3^; common causes of severe acute hepatitis among children include hepatotropic viruses, other infectious pathogens, and exposure to toxins.^4^ An early hypothesis regarding the etiology of the observed condition was that a cofactor affected young children with adenovirus infection, which would be mild in normal circumstances, triggering a more severe infection or immune‐mediated liver damage.^5^ Details of the outbreak and its investigation have been previously published.^6^, ^7^, ^8^, ^9^, ^10^, ^11^, ^12^

The European Centre for Disease Control and Prevention (ECDC) and WHO Europe recommended that public health authorities should communicate with pediatricians, general practitioners, and other medical specialists to inform about the need for active case finding and reporting of new cases.^5^, ^13^ ECDC also suggested case reporting to the European Surveillance System (TESSy).^14^

In Germany, severe acute hepatitis and pediatric acute liver failure (pALF) of unknown etiology among children were not under routine surveillance before 2022. According to the Infection Protection Act, medical doctors must notify suspected cases of new life‐threatening diseases to local public health authorities.^15^

Children and adolescents with pALF younger than 18 years are routinely referred to and treated in specialized pediatric hepatological centers (pediatric liver transplant centers), where they are evaluated for receiving a liver transplant if needed. Diagnostic work‐up is similar in all major pediatric liver transplant centers in Germany, including a wide range of noninfectious as well as infectious causes (including adenovirus). In Germany, eight such centers exist.

To investigate whether Germany was affected by the increase of severe acute hepatitis cases of unknown etiology among previously healthy children, a team at the Robert Koch Institute (RKI) in collaboration with the working group of pediatric liver transplant centers of the German Pediatric Society of Gastroenterology and Nutrition assessed whether more cases than expected occurred in Germany in 2022.

## 2 METHODS

For the investigation of this potential outbreak, we conducted active case finding, set up active surveillance, and used secondary hospital discharge data to identify cases and assess if the numbers were higher than expected.

### 2.1 Ethics statement

Ethical approval was not obtained for the investigation as it was covered by the German Infection Prevention Act. We only used aggregated data from clinical centers and routinely available data from the federal statistical office.

### 2.2 Active case finding and surveillance

An investigation team at RKI was established on April 7, 2022. In the acute phase of the outbreak (April and May 2022), pediatric and hepatologic societies and public health authorities in Germany were informed on April 7, April 11, April 26, April 29, and May 11, 2022 about the reports of severe acute hepatitis cases of unknown etiology in children by other countries and asked as to whether clinicians in Germany experienced an increase since January 2022.

We developed a case definition adapted from ECDC's case definition.^5^ A probable case was defined as

1. a person who is 16 years or younger presenting with severe acute hepatitis with onset after January 1, 2022,
2. with elevated serum transaminases greater than 500 IU/L (aspartate transaminase or alanine transaminase),
3. with adenovirus detection or unexplained etiology.

Cases with known etiology (e.g., caused by toxins, by a specific metabolic, hereditary, or autoimmune condition) should not be reported.^16^

For this analysis, we further defined the term “fulminant” pALF as severely impaired liver function that resulted in listing for a liver transplant, undergoing a liver transplant or death. This definition was established in contrast to the internationally accepted definition of “pALF”^4^, ^17^, ^18^, ^19^ to enable the participating pLTxCs to timely report retrospective data.

From April 2022 onward medical doctors were asked to notify suspected cases of severe acute hepatitis that met the case definition to the local public health authorities, according to the Infection Protection Act.

Active surveillance was set up from May 2022 to May 2023 to enable quick detection of a possible increase in the number of cases. We asked all pediatric liver transplant centers in Germany (*n* = 8) to prospectively report monthly numbers of patients with severe acute hepatitis with adenovirus detection or unclear etiology with and without fulminant pALF. To establish a baseline, centers were asked to provide retrospective numbers of patients with fulminant pALF (as defined above) of unknown etiology in 2017–2021 and the first quarter of 2022. Retrospective numbers of patients with severe acute hepatitis of unknown etiology without pALF that did not result in the need for listing, liver transplantation, or death could not be reported with reasonable additional effort by the centers and were thus not available for our analysis.

To test for statistical differences between 2017 and 2021 and 2022, we used a two‐sample Poisson test, with a significance level of *p* = 0.05.

### 2.3 Analysis of hospital discharge diagnoses and procedures

To increase sensitivity to possibly occurring milder courses of disease treated in non‐specialized hospitals, we analyzed data on hospital discharge diagnoses and procedures from the German federal statistical office.

All German hospitals submit the main diagnosis of each inpatient as four‐digit International Classification of Diseases, Tenth Revision (ICD‐10) diagnostic code to regional health authorities and provide age (in years) and sex of the patient as well as other information (e.g., length of hospital stay and diagnosis related group [DRG] procedure). Data are cleared by regional health authorities, and then transferred to the German federal statistical office (DESTATIS). DESTATIS publishes frequencies of ICD‐10 codes, completely anonymized, stratified by age group (<1, 1–4, 5–9, 10–14, 15–17, 18–19 years, etc.) and sex. Data are publicly available online with a 1‐year delay.^20^

Following ECDC's protocol for assessment of exceedances around severe acute cases of hepatitis of unknown origin in children in EU/EEA countries,^21^ we selected 11 hepatological ICD‐10 codes indicating hepatitis other than viral hepatitis A, B, C, or E infection for the analysis of inpatient numbers. To be as sensitive as possible, we added “Autoimmune hepatitis” (K75.4)^22^ to the list of ICD‐10 codes for inclusion of pediatric inpatients:

1. R17.0 Hyperbilirubinemia, with or without jaundice, not elsewhere classified
2. B17.8 Other specified acute viral hepatitis
3. B17.9 Acute viral hepatitis, unspecified
4. B19.0 Unspecified viral hepatitis with hepatic coma
5. B19.9 Unspecified viral hepatitis without hepatic coma
6. K71.6 Toxic liver disease with hepatitis, not elsewhere classified
7. K72.0 Acute and subacute hepatic failure
8. K72.9 Hepatic failure, unspecified
9. K75.2 Nonspecific reactive hepatitis
10. K75.4 Autoimmune hepatitis
11. K75.9 Inflammatory liver disease, unspecified
12. Z94.4 Liver transplant status.

We also analyzed the DRG procedure “liver transplant” (OPS‐5‐504) by age group and year. Our analysis of inpatients was limited to the age groups <1, 1–4, 5–9, 10–14, and 15–17 to correspond best to our case definition mentioned above.

As only the main hospital discharge diagnoses are published, secondary or contributing diagnostic codes cannot be considered.

To detect any temporal peaks for pediatric inpatients, we obtained and analyzed nonpublic data from DESTATIS stratified by month of hospital admission.

We compared annual numbers of inpatients for each ICD‐10 code separately for the years 2015 to 2022. For stratification, annual numbers of inpatients with selected ICD‐10 codes for the years 2015 to 2022 were combined. We stratified by

1. disease severity:

- hepatic failure: ICD‐10 codes K72.0, K72.9, Z94.4
- hepatitis: ICD‐10 codes B17.8, B17.9, B19.0, B19.9, K71.6, K75.2, K75.4, K75.9
- hyperbilirubinemia: ICD‐10 code R17.0

2. age groups: <1, 1–4, 5–9, 10–14, 15–17 years
3. month of hospital admission.

As the ICD‐10 code R17.0 (hyperbilirubinemia with jaundice, not elsewhere classified) was introduced in Germany only in 2019, annual and monthly numbers of inpatients diagnosed with R17.0 were analyzed separately for the years 2019 to 2022.

We used a two‐sample Poisson test to test for statistical differences between 2015 to 2021 and 2022. We chose a significance level of *p* = 0.05.

We used MS Excel (version Excel 2405), R version 4.31, and RStudio version 2023.06.2.61 for data analysis.

## 3 RESULTS

### 3.1 Active case finding and surveillance

The inquiries made to clinical societies in April and May 2022 did not reveal an unusual increase in patients with severe acute hepatitis with adenovirus detection or unexplained etiology in pediatric liver centers in Germany since January 2022.

#### 3.1.1 I. cases reported under the infection protection act

Under the Infection Protection Act, a total of 22 cases (mean age 8.1 years, range 0–16 years) were notified from April 19, 2022 to March 2, 2023, which were very heterogeneous regarding diagnostic tests performed and previous/underlying diseases. Thirteen cases (59%) were notified between April 25, 2022 and May 22, 2022. Disease onsets ranged from January 19, 2022 to February 13, 2023. Supplement S1 shows notified cases by week of disease onset. There were no baseline data available for comparison.

#### 3.1.2 II. Case numbers reported by pediatric liver transplant centers

All eight pediatric liver transplant centers participated in the active surveillance.

#### 3.1.3 II.a Number of severe acute hepatitis of unknown etiology with fulminant pALF

From May 2022 to May 2023, the eight centers reported a total of nine severe acute hepatitis cases of unknown etiology with fulminant pALF compared to 5–14 fulminant pALF cases annually in 2017–2021 (*p* = 0.53). In the first quarter of 2022, two fulminant pALF cases were reported retrospectively. Adenovirus was detected in none of the cases.

One center reported three fulminant pALF cases of unknown etiology, listed for Tx, in April and May 2022 before the prospective survey started. In this center, the number of cases in quarter 2 of 2022 (*n* = 4) was higher than in the years 2017–2021 (range 0–3, median: 1) with borderline statistical significance (*p* = 0.048). The total number of cases in quarter 1–4 of 2022 (*n* = 4) was not statistically higher than in the years 2017–2021 (*p* = 0.19). Adenovirus Type 9 was detected in one of the cases. The phenotype was similar in most cases: a preceding episode with gastrointestinal symptoms and manifestation of hepatitis ~2–3 weeks after gastroenteritis. All four cases responded well to steroids and/or plasmapheresis. None of these cases needed to be transplanted or died.

#### 3.1.4 II.b Number of severe acute hepatitis of unknown etiology without fulminant pALF

From May 2022 to May 2023, the eight centers reported a total of 26 severe acute hepatitis cases without fulminant pALF. Adenovirus was detected in four (blood and/or stool) and suspected (test results not entirely conclusive) in two of the cases. Out of the 26 reported cases, five patients received plasmapheresis and eight were treated with steroids. There were no baseline data available for comparison.

### 3.2 Analysis of hospital discharge diagnoses and procedures

A total of 373 pediatric inpatients were diagnosed with any of the selected main diagnoses ICD‐10 codes B17.8, B17.9, B19.0, B19.9, K71.6, K72.0, K72.9, K75.2, K75.4, K75.9, and Z94.4 in 2022, compared to an annual range of 333–422 in 2015 to 2021 (median: 385; *p* = 0.63). The number of inpatients with a diagnosis of hyperbilirubinemia (R17.0) was 322, compared to an annual range of 298–353 in 2019 to 2021 (median: 332, *p* = 0.61). There were 85 liver transplants in patients aged 0–17 years in 2022, compared to an annual range of 91–114 in 2015 to 2021 (median: 97; *p* = 0.94).

Analysis of specific ICD‐10 codes revealed no statistically significant increases in total number of inpatients aged 0–17 years (see Supplement S2 for comprehensive table of ICD‐10 code numbers by year and age group). When stratifying by age groups, there were statistically significant increases in the 1‐ to 4‐year olds diagnosed with “acute viral hepatitis, unspecified” (ICD‐10 code B17.9; *n* = 9 inpatients in 2022, yearly median for 2015–2021: 4/year, range: 1–8, *p* = 0.035) as well as the 5‐ to 9‐year olds and the 15‐ to 17‐year olds diagnosed with “hyperbilirubinemia, with jaundice, not elsewhere classified” (*n* = 12 inpatients 5–9 years in 2022, yearly median for 2019–2021 = 5/year, range: 4–8, *p* = 0.039 and *n* = 25 inpatients 15–17 years in 2022, yearly median for 2019–2021: 12/year, range: 12–19, *p* = 0.021).

**Figure 1:**
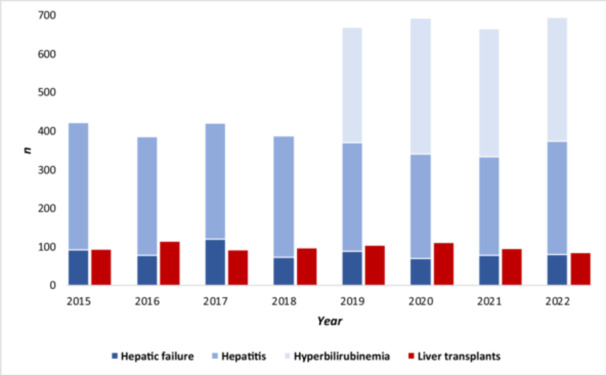
Annual numbers of inpatients aged 0–17 years diagnosed with any of the selected hospital discharge diagnoses (stacked blue columns) and liver transplants (red column), by year and disease severity (Hepatic failure: ICD‐10 codes K72.0, K72.9, Z94.4; Hepatitis: ICD‐10 codes B17.8, B17.9, B19.0, B19.9, K71.6, K75.2, K75.4, K75.9; Hyperbilirubinemia: ICD‐10 code R17.0, available only from 2019 onward; liver transplant: DRG OPS‐5‐504), according to hospital discharge data and operations and procedures on inpatients from the German federal statistical office (DESTATIS), Germany, 2015–2022. DRG, diagnosis related group; ICD‐10, International Classification of Diseases, Tenth Revision.

**Figure 2:**
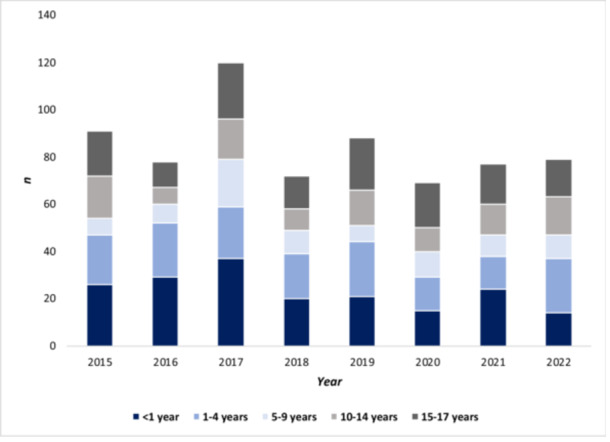
Annual numbers of inpatients aged 0–17 years with hepatic failure diagnoses (ICD‐10 codes K72.0, K72.9, Z94.4), by year and age group, according to hospital discharge data from the German federal statistical office (DESTATIS), Germany, 2015–2022. ICD‐10, International Classification of Diseases, Tenth Revision.

**Figure 3:**
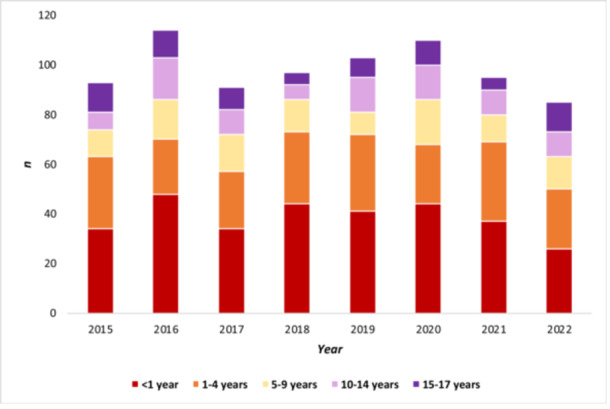
Annual numbers of liver transplants (DRG OPS‐5‐504) in patients aged 0–17 years, by year and age group, according to operations and procedures on inpatients from the German federal statistical office (DESTATIS), Germany, 2015–2022. DRG, diagnosis related group.

There was no increase in the number of cases stratified by disease severity in inpatients aged 0–17 years in 2022: hyperbilirubinemia^a^ (*p* = 0.61), hepatitis (*p* = 0.52), hepatic failure (*p* = 0.75), nor liver transplants (*p* = 0.94) (Figure 1). Further stratifying by age groups, hepatic failure and need for a liver transplant did not disproportionately affect any age group in 2022, especially not the age group of 1‐ to 4‐year‐olds (*p* = 0.26*)* as reported by United Kingdom, compared to previous years (Figures 2 and 3).

**Figure 4:**
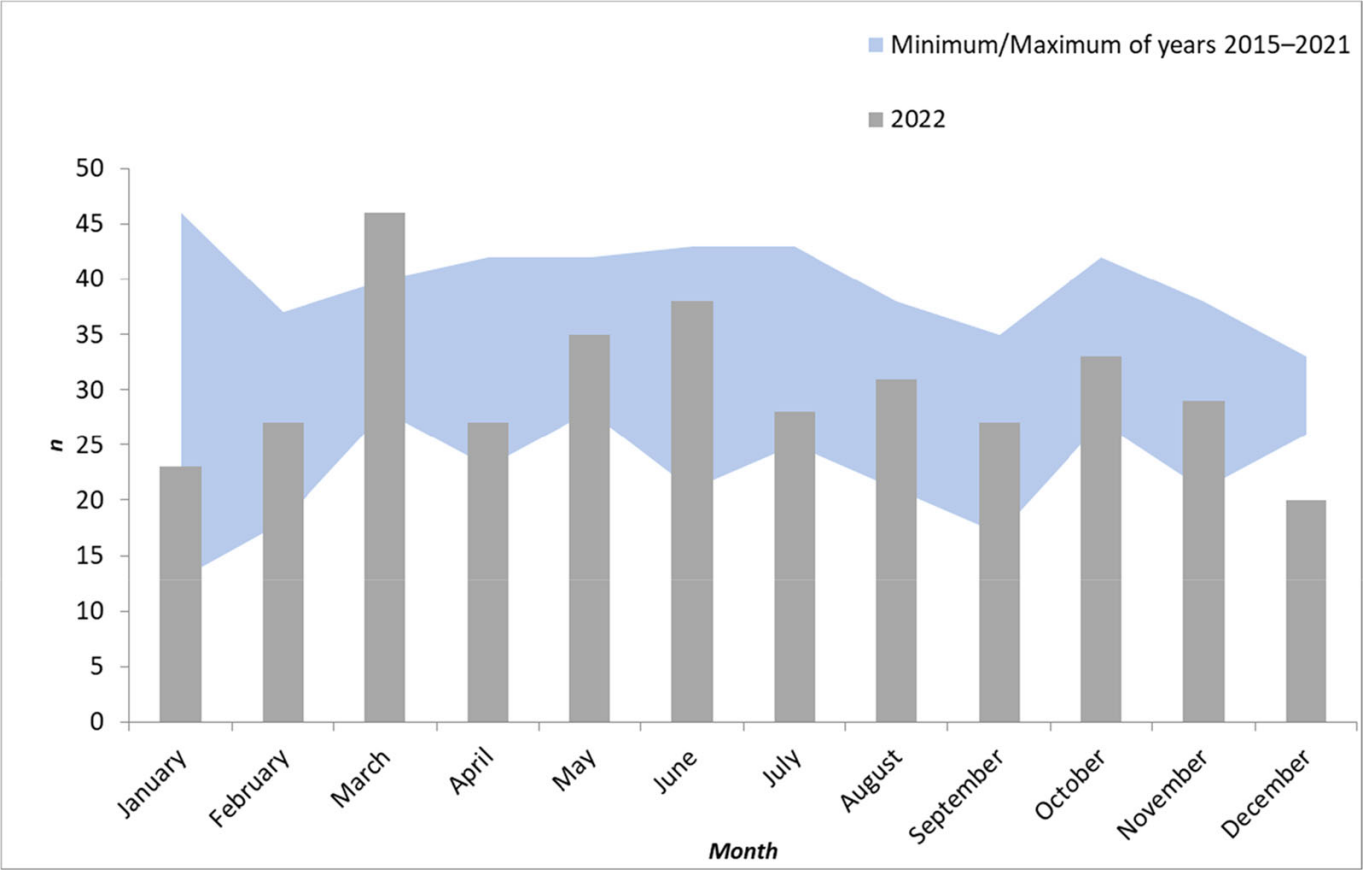
Monthly numbers of inpatients aged 0–17 years diagnosed with any of the selected discharge diagnoses (ICD‐10 codes B17.8, B17.9, B19.0, B19.9, K71.6, K72.0, K72.9, K75.2, K75.4, K75.9, Z94.4), (2022 [bars] vs. range 2015–2021 [area]), by month of hospital admission, according to hospital discharge data upon special request from the German federal statistical office (DESTATIS), Germany, 2015–2022. ICD‐10; International Classification of Diseases, Tenth Revision.

Stratifying by month of hospital admission, number of admitted inpatients 0–17 years in March 2022 (*n* = 46) was higher than the respective numbers in 2015–2021 (range: 28–40 patients; median = 33; *p* = 0.032). Admission numbers in remaining months did not reveal any unexpected increases (Figure 4). Stratification of admission numbers in March by disease severity did not reveal a significant increase in number of hepatic failure diagnoses (*p* = 0.11). Further stratification, for example, by age group or specific ICD‐10 codes, was not possible due to data protection restrictions related to low inpatient numbers per strata.

## 4 DISCUSSION

According to the available data and assessment of clinical experts,^23^, ^24^ there was no apparent increase of severe acute hepatitis cases with adenovirus detection or unknown etiology resulting in fulminant pALF in children and adolescents in Germany as observed in the United Kingdom in 2022. This perception was also shared by most pediatric hepatologists in Germany.

Our assessment was based on two separate data sources: (a) cases of severe acute hepatitis with fulminant pALF as defined in the methods section treated in pediatric liver transplant centers with and without the need for liver transplantation and (b) hospital discharge data from pediatric inpatients of all German hospitals. For cases notified under the Infection Prevention Act, there were no baseline data available for comparison. Temporal clustering of cases could be due to increased awareness among physicians in April and May 2022.

Our results show that numbers of severe acute hepatitis cases of unknown etiology with fulminant pALF as well as numbers of pediatric inpatients with specific ICD‐10 codes remained stable in 2022 compared to previous years. Even though we found statistically significant increases when stratifying separate ICD‐10 codes by age groups and when stratifying aggregated counts by admission month, the detected differences were small in absolute numbers (≈6 inpatients) and did not affect very severe stages of disease, that is, numbers of inpatients with fulminant liver failure or number of liver transplants. Clinical significance of this finding thus remains questionable.

Our results are in line with other countries' investigations that did not find a clear increase in severe acute hepatitis cases of unknown etiology in children in 2022,^22^, ^25^, ^26^, ^27^ supporting regional differences. A recent publication from two of the eight pediatric liver transplant centers in Germany described an increase in annual incidence per center for the years 2019–2022 compared to a historic cohort until 2018, with no particular increase in 2022.^28^ One out of eight German pediatric liver transplant centers observed a local cluster with a proportionate increase in severe acute hepatitis cases of unknown etiology in quarter 2 of 2022, with no need for liver transplants and no deaths.^29^ Considering data from all eight centers, no increase was seen. Potential direct or indirect effects of the COVID‐19 pandemic (e.g., rebound phenomena of viral infections) and of allocation/referral of severely ill patients need to be further investigated, considering cases of all centers.

Our investigation has several limitations. Estimating typical background disease activity to compare baseline numbers of severe acute hepatitis cases with prospectively collected case numbers proved challenging due to potential heterogeneity of diagnostic procedures over time and across pediatric centers. It is noteworthy that the diagnostic work‐up of children with pALF is similar in all pediatric liver transplant centers mentioned herein.

Baseline data for evaluation of cases reported under the Infection Protection Act and cases of severe acute hepatitis without liver failure were not available for our analysis. Reporting of case numbers by pediatric liver transplant centers only started in May 2022, which means a data gap for April 2022, where the assessment solely relies on the clinical experts. Our definition of fulminant pALF cases included listing for liver transplant, receiving a liver transplant, or death, which limited the data to the most severe cases, and we might have missed clinical cases with less severe course. As for the hospital discharge data, the 12 selected ICD‐10 codes most likely also include inpatients that would not meet our case definition as data on secondary diagnoses of inpatients were not available in DESTATIS, and we could thus not exclude any cases with known etiology. Despite these limitations, our data provide important information in the form of “best available evidence”,^30^ and are in line with data analyses as suggested by ECDC and WHO.

## 5 CONCLUSION

To conclude, we could not identify an increase of severe acute hepatitis cases with adenovirus detection or unknown etiology with fulminant liver failure in children and adolescents in Germany as observed in the United Kingdom in 2022. We show that close collaboration between clinical and public health actors is essential when investigating a condition without an existing surveillance system already in place.^31^ This study aims to raise awareness of the importance of early recognition of unusual symptom patterns (e.g., jaundice), strengthened communication between clinicians and public health authorities, and the potential of syndromic surveillance to support timely responses—ultimately benefiting children presenting with signs such as elevated liver enzymes. A syndromic surveillance system ^32^ in pediatric emergency departments that is currently being established in Germany ^33^ will facilitate the analysis of routinely collected and anonymized emergency department data on syndromes, such as “jaundice” or “gastroenteritis,” in real‐time for surveillance and public health research. This may support future outbreak detection, improve rapid information to health care professionals and facilitate prompt investigations. We further recommend more timely access to hospital discharge data including secondary diagnoses.

## CONFLICT OF INTEREST STATEMENT

The authors declare no conflicts of interest.

## Supporting information


**Supplement 1:** Number of notified cases of acute severe hepatitis of unknown etiology under the Infection Protection Act by months (we used date of disease onset where available (n = 17 cases), then date of notification (n = 5 cases)). **Supplement 2:** Numbers of inpatients 0–17 years with selected International Classification of Diseases, Tenth Revision (ICD‐10) codes and number of liver transplants, by year and age group, according to hospital discharge diagnoses and operations and procedures on inpatients from the German federal statistical office (DESTATIS), Germany, 2015–2022.

## Footnotes

a As the ICD‐10 code R17.0 for hyperbilirubinemia was only introduced in 2019, we compared the mean for the years 2019–2021 with 2022.
