# Supplementary material for: No apparent increase in cases of severe acute hepatitis of unknown etiology with fulminant liver failure in children in Germany, 2022
Source: JPGN Rep. 2025 Sep 12;6(4):498–507. doi: 10.1002/jpr3.70083 (PMC12611568; doi:10.1002/jpr3.70083)
Supplement: Supplementary file 1 — Supplement 1: Number of notified cases of acute severe hepatitis of unknown etiology under the Infection Protection Act by months (we used date of disease onset where available (n = 17 cases), then date of notification (n = 5 cases)). Supplement 2: Numbers of inpatients 0–17 years with selected International Classification of Diseases, Tenth Revision (ICD‐10) codes and number of liver transplants, by year and age group, according to hospital discharge diagnoses and operations and procedures on inpatients from the German federal statistical office (DESTATIS), Germany, 2015–2022. [file JPR3-6-498-s001.docx]

**Supplemental Digital Content**

**
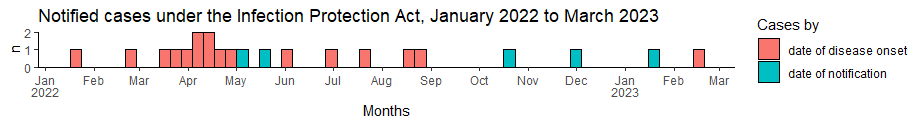
**

**Supplement 1: Number of notified cases of acute severe hepatitis of unknown etiology under the Infection Protection Act by months (we used date of disease onset where available (n=17 cases), then date of notification (n=5 cases)).**

**Supplement 2: Numbers of inpatients 0**–**17 years with selected ICD-10 codes and number of liver transplants, by year and age group, according to hospital discharge diagnoses and operations and procedures on inpatients from DESTATIS, Germany, 2015**–**2022**

| **R17.0 Hyperbilirubinemia, with jaundice, not elsewhere classified** | | | | | | |
| --- | --- | --- | --- | --- | --- | --- |
| **Year** | **<1 year** | **1-4 years** | **5-9 years** | **10-14 years** | **15-17 years** | **Total** |
| 2019 | 262 | 5 | 5 | 14 | 12 | 298 |
| 2020 | 305 | 8 | 4 | 17 | 19 | 353 |
| 2021 | 297 | 3 | 8 | 12 | 12 | 332 |
| 2022 | 267 | 4 | 12 | 14 | 25 | 322 |
|  |  |  |  |  |  |  |
| **B17.8 Other specified acute viral hepatitis** | | | | | | |
| **Year** | **<1 year** | **1-4 years** | **5-9 years** | **10-14 years** | **15-17 years** | **Total** |
| 2015 | 1 | 4 | 1 | 7 | 4 | 17 |
| 2016 | 0 | 1 | 1 | 2 | 10 | 14 |
| 2017 | 0 | 0 | 2 | 5 | 3 | 10 |
| 2018 | 5 | 4 | 3 | 2 | 3 | 17 |
| 2019 | 0 | 4 | 0 | 2 | 8 | 14 |
| 2020 | 1 | 3 | 4 | 3 | 1 | 12 |
| 2021 | 2 | 5 | 3 | 1 | 3 | 14 |
| 2022 | 1 | 5 | 3 | 6 | 2 | 17 |
|  |  |  |  |  |  |  |
| **B17.9 Acute viral hepatitis, unspecified** | | | | | | |
| **Year** | **<1 year** | **1-4 years** | **5-9 years** | **10-14 years** | **15-17 years** | **Total** |
| 2015 | 1 | 8 | 12 | 8 | 7 | 36 |
| 2016 | 2 | 2 | 5 | 4 | 7 | 20 |
| 2017 | 3 | 1 | 7 | 5 | 6 | 22 |
| 2018 | 3 | 6 | 5 | 6 | 7 | 27 |
| 2019 | 2 | 3 | 5 | 6 | 5 | 21 |
| 2020 | 1 | 4 | 1 | 4 | 7 | 17 |
| 2021 | 5 | 4 | 8 | 6 | 0 | 23 |
| 2022 | 1 | 9 | 9 | 3 | 7 | 29 |
|  |  |  |  |  |  |  |
| **B19.0 Unspecified viral hepatitis with hepatic coma** | | | | | | |
| **Year** | **<1 year** | **1-4 years** | **5-9 years** | **10-14 years** | **15-17 years** | **Total** |
| 2015 | 0 | 0 | 0 | 0 | 0 | 0 |
| 2016 | 0 | 0 | 0 | 0 | 0 | 0 |
| 2017 | 0 | 0 | 0 | 0 | 0 | 0 |
| 2018 | 0 | 0 | 0 | 0 | 0 | 0 |
| 2019 | 0 | 0 | 0 | 0 | 0 | 0 |
| 2020 | 0 | 0 | 0 | 0 | 0 | 0 |
| 2021 | 0 | 0 | 0 | 0 | 0 | 0 |
| 2022 | 0 | 0 | 0 | 0 | 0 | 0 |
| **B19.9 Unspecified viral hepatitis without hepatic coma** | | | | | | |
| **Year** | **<1 year** | **1-4 years** | **5-9 years** | **10-14 years** | **15-17 years** | **Total** |
| 2015 | 1 | 2 | 2 | 5 | 3 | 13 |
| 2016 | 0 | 1 | 2 | 0 | 5 | 8 |
| 2017 | 1 | 2 | 3 | 4 | 3 | 13 |
| 2018 | 2 | 2 | 3 | 4 | 2 | 13 |
| 2019 | 0 | 0 | 0 | 1 | 6 | 7 |
| 2020 | 1 | 3 | 2 | 3 | 8 | 17 |
| 2021 | 1 | 3 | 0 | 0 | 3 | 7 |
| 2022 | 1 | 3 | 4 | 2 | 4 | 14 |
|  |  |  |  |  |  |  |
| **K71.6 Toxic liver disease with hepatitis, not elsewhere classified** | | | | | | |
| **Year** | **<1 year** | **1-4 years** | **5-9 years** | **10-14 years** | **15-17 years** | **Total** |
| 2015 | 0 | 3 | 2 | 1 | 7 | 13 |
| 2016 | 0 | 3 | 0 | 3 | 5 | 11 |
| 2017 | 0 | 0 | 3 | 4 | 5 | 12 |
| 2018 | 1 | 0 | 3 | 3 | 4 | 11 |
| 2019 | 1 | 1 | 1 | 1 | 1 | 5 |
| 2020 | 1 | 1 | 0 | 2 | 5 | 9 |
| 2021 | 1 | 0 | 1 | 0 | 5 | 7 |
| 2022 | 0 | 0 | 2 | 3 | 2 | 7 |
|  |  |  |  |  |  |  |
| **K72.0 Acute and subacute hepatic failure** | | | | | | |
| **Year** | **<1 year** | **1-4 years** | **5-9 years** | **10-14 years** | **15-17 years** | **Total** |
| 2015 | 22 | 19 | 7 | 17 | 17 | 82 |
| 2016 | 24 | 21 | 6 | 7 | 11 | 69 |
| 2017 | 33 | 21 | 18 | 14 | 20 | 106 |
| 2018 | 13 | 16 | 9 | 5 | 10 | 53 |
| 2019 | 14 | 20 | 5 | 8 | 18 | 65 |
| 2020 | 13 | 14 | 10 | 8 | 15 | 60 |
| 2021 | 22 | 13 | 6 | 11 | 16 | 68 |
| 2022 | 12 | 20 | 6 | 15 | 15 | 68 |
|  |  |  |  |  |  |  |
| **K72.9 Hepatic failure, unspecified** | | | | | | |
| **Year** | **<1 year** | **1-4 years** | **5-9 years** | **10-14 years** | **15-17 years** | **Total** |
| 2015 | 4 | 2 | 0 | 1 | 2 | 9 |
| 2016 | 5 | 2 | 2 | 0 | 0 | 9 |
| 2017 | 3 | 1 | 2 | 3 | 4 | 13 |
| 2018 | 7 | 3 | 1 | 4 | 4 | 19 |
| 2019 | 7 | 3 | 1 | 7 | 4 | 22 |
| 2020 | 2 | 0 | 1 | 2 | 4 | 9 |
| 2021 | 2 | 1 | 3 | 2 | 1 | 9 |
| 2022 | 2 | 2 | 4 | 1 | 1 | 10 |
|  |  |  |  |  |  |  |
| **K75.2 Nonspecific reactive hepatitis** | | | | | | |
| **Year** | **<1 year** | **1-4 years** | **5-9 years** | **10-14 years** | **15-17 years** | **Total** |
| 2015 | 1 | 5 | 4 | 11 | 3 | 24 |
| 2016 | 1 | 6 | 2 | 3 | 12 | 24 |
| 2017 | 1 | 3 | 2 | 2 | 4 | 12 |
| 2018 | 1 | 3 | 5 | 9 | 6 | 24 |
| 2019 | 1 | 4 | 0 | 5 | 0 | 10 |
| 2020 | 1 | 1 | 5 | 6 | 5 | 18 |
| 2021 | 0 | 2 | 1 | 6 | 2 | 11 |
| 2022 | 2 | 2 | 4 | 4 | 6 | 18 |
|  |  |  |  |  |  |  |
| **K75.4 Autoimmune hepatitis** | | | | | | |
| **Year** | **<1 year** | **1-4 years** | **5-9 years** | **10-14 years** | **15-17 years** | **Total** |
| 2015 | 0 | 11 | 27 | 76 | 57 | 171 |
| 2016 | 0 | 11 | 18 | 60 | 75 | 164 |
| 2017 | 2 | 19 | 23 | 63 | 64 | 171 |
| 2018 | 0 | 9 | 11 | 78 | 69 | 167 |
| 2019 | 1 | 17 | 28 | 73 | 67 | 186 |
| 2020 | 0 | 9 | 19 | 69 | 52 | 149 |
| 2021 | 0 | 10 | 21 | 57 | 69 | 157 |
| 2022 | 0 | 5 | 16 | 80 | 62 | 163 |
|  |  |  |  |  |  |  |
| **K75.9 Inflammatory liver disease, unspecified** | | | | | | |
| **Year** | **<1 year** | **1-4 years** | **5-9 years** | **10-14 years** | **15-17 years** | **Total** |
| 2015 | 2 | 9 | 17 | 19 | 10 | 57 |
| 2016 | 7 | 7 | 10 | 23 | 19 | 66 |
| 2017 | 4 | 2 | 12 | 16 | 27 | 61 |
| 2018 | 11 | 8 | 8 | 13 | 16 | 56 |
| 2019 | 4 | 7 | 6 | 11 | 10 | 38 |
| 2020 | 5 | 8 | 10 | 10 | 16 | 49 |
| 2021 | 1 | 4 | 3 | 18 | 11 | 37 |
| 2022 | 2 | 5 | 8 | 12 | 19 | 46 |
|  |  |  |  |  |  |  |
| **Z94.4 Liver transplant status (post liver transplant)** | | | | | | |
| **Year** | **<1 year** | **1-4 years** | **5-9 years** | **10-14 years** | **15-17 years** | **Total** |
| 2015 | 0 | 0 | 0 | 0 | 0 | 0 |
| 2016 | 0 | 0 | 0 | 0 | 0 | 0 |
| 2017 | 1 | 0 | 0 | 0 | 0 | 1 |
| 2018 | 0 | 0 | 0 | 0 | 0 | 0 |
| 2019 | 0 | 0 | 1 | 0 | 0 | 1 |
| 2020 | 0 | 0 | 0 | 0 | 0 | 0 |
| 2021 | 0 | 0 | 0 | 0 | 0 | 0 |
| 2022 | 0 | 1 | 0 | 0 | 0 | 1 |
|  |  |  |  |  |  |  |
| **OPS-5-504 Liver transplants** | | | | | | |
| **Year** | **<1 year** | **1-4 years** | **5-9 years** | **10-14 years** | **15-17 years** | **Total** |
| 2015 | 34 | 29 | 11 | 7 | 12 | 93 |
| 2016 | 48 | 22 | 16 | 17 | 11 | 114 |
| 2017 | 34 | 23 | 15 | 10 | 9 | 91 |
| 2018 | 44 | 29 | 13 | 6 | 5 | 97 |
| 2019 | 41 | 31 | 9 | 14 | 8 | 103 |
| 2020 | 44 | 24 | 18 | 14 | 10 | 110 |
| 2021 | 37 | 32 | 11 | 10 | 5 | 95 |
| 2022 | 26 | 24 | 13 | 10 | 12 | 85 |
